# Structure, function and druggability of the African trypanosome flagellum

**DOI:** 10.1002/jcp.30778

**Published:** 2022-05-26

**Authors:** Julia Sáez Conde, Samuel Dean

**Affiliations:** ^1^ Division of Biomedical Sciences, Warwick Medical School University of Warwick Coventry UK

**Keywords:** African trypanosomiasis, chemotherapy, flagellum, model organism, parasite, trypanosome

## Abstract

African trypanosomes are early branching protists that cause human and animal diseases, termed trypanosomiases. They have been under intensive study for more than 100 years and have contributed significantly to our understanding of eukaryotic biology. The combination of conserved and parasite‐specific features mean that their flagellum has gained particular attention. Here, we discuss the different structural features of the flagellum and their role in transmission and virulence. We highlight the possibilities of targeting flagellar function to cure trypanosome infections and help in the fight to eliminate trypanosomiases.

## Graphical abstract

African trypanosomes are flagellated protists that cause human and animal diseases, termed trypanosomiases. Here, we discuss the different structural features of the flagellum and their role in transmission and virulence. We highlight the possibilities of targeting flagellar function to cure trypanosome infections and help in the fight to eliminate trypanosomiases.

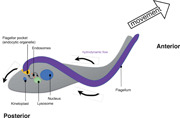

## 1 TRYPANOSOMES AND AFRICAN TRYPANOSOMIASIS

African trypanosomes are parasitic kinetoplastids best known for causing Human African Trypanosomiasis (HAT), also known as sleeping sickness, and nagana in cattle. Trypanosomes are transmitted between mammalian hosts via the tsetse fly in sub‐Saharan African. Nagana is caused by several trypanosome subspecies (Mehlitz & Molyneux, 2019) and causes decreases in meat and milk yields, contributing to keeping rural farmers poor and economic hardship (Ebhodaghe et al., 2018).

HAT is a lethal disease caused by African trypanosome subspecies *Trypanosome brucei rhodesiense* and *Trypanosoma brucei gambiense*. HAT begins with a bite from an infected tsetse fly and the first signs of infection start 2–3 days after inoculation with a chancre at the site of the tsetse bite and inflamed lymph nodes at the back of the neck ('Winterbottom's sign') (Steverding, 2008).

The early disease is characterised by fever and headaches and is often misdiagnosed. It is in this 'haemolymphatic phase' that the parasites proliferate and spread in the blood and lymphatic system. In the late stage of the disease, the encephalitic phase, parasites cross the blood–brain barrier and enter the central nervous system causing neurological symptoms, including the sleeping cycle abnormalities from which the disease is named (P. G. E. Kennedy, 2019). If left untreated, HAT can progress onto coma followed by death (Croft et al., 2007; P. G. Kennedy, 2013; Wengert et al., 2014).

## 2 TRYPANOSOME FLAGELLA AND THE CELL CYCLE

**Figure 1:**
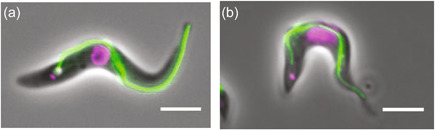
Trypanosomes are well‐designed swimmers. Epifluorescence live cell microscopy of (a) an insect stage and (b) a bloodstream stage trypanosome expressing a fluorescently tagged flagellum protein (green) and stained with Hoechst to visualise the DNA (magenta). Note the flagellum is attached to the cell body for most of its length. Trypanosomes swim left to right as shown here. Scale bar = 5 μm. Figure adapted from Dean & Sunter (2020).

Trypanosome cells are 18–32 μm long and 1.5–3 μm wide and have a ‘teardrop' shape (Bruce, 1911). Their flagellum is bound to the cell body for most of its length and performs a 180° turn around the body (Bargul et al., 2016). The basal body defines the proximal end of the flagellum and is embedded in the cytoplasm alongside an immature 'pro‐basal body'. The distal flagellum tip is ‘free’, extending somewhat beyond the anterior of the cell body (Figure 1).

New flagellum assembly occurs next to the old and plays an intricate role in the cell cycle of the trypanosome (Robinson et al., 1995; Sherwin & Gull, 1989). Growth of a new flagellum initiates from the newly formed basal body after pro‐basal body reorientation and maturation. As the new flagellum extends, the new basal body moves towards the posterior of the cell, which is crucial for placement of the plasma membrane invagination that occurs during cytokinesis ('cytokinetic groove') and proper inheritance of organelles at cell division. New pro‐basal bodies start forming next to the new and old mature basal bodies before cytokinesis ends (Sherwin & Gull, 1989), resulting in daughter cells inheriting a basal body pair semi‐conservatively (Wheeler et al., 2019).

Trypanosome flagella are built by a highly conserved kinesin and dynein transport system called intra‐flagellar transport (IFT) that imports flagellar components from the cytoplasm and adds to the distal end of the flagellum for growth. When the flagellum reaches a defined length, it ‘locks’ and growth arrests (Bertiaux et al., 2018). This locking requires a CEP164 homologue at the flagellum base and may involve negative regulation of IFT function (Atkins et al., 2021). However, many questions remain unanswered, such as how the cells measure the flagellum length, the biochemical nature of the locking mechanism, and the precise role for IFT in this process.

## 3 THE MICROTUBULE‐BASED FLAGELLUM IS CANONICAL WITH LINEAGE‐SPECIFIC INNOVATIONS

**Figure 2:**
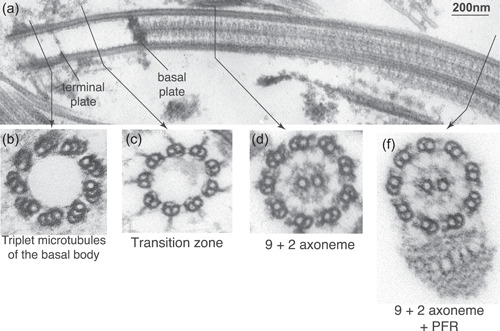
The trypanosome flagellum has the canonical microtubule domains. (a) A longitudinal transmission electron microscopy (TEM) section through a detergent‐treated trypanosome cell reveals the three domains of the flagellum delineated by terminal and basal plates. Cross‐sections through the flagellum reveal (b) the basal body triplet microtubules (9 + 0), (c) the transition zone doublets (9 + 0), and (d) the proximal and (e) distal flagellum (9 + 2). Note the PFR that starts approximately 1 µm from the flagellum base. Figure adapted from Vaughan et al. (2006). PFR, paraflagellar rod.

The trypanosome flagellum has the canonical microtubule arrangement found in motile flagella across eukaryotic life (Figure 2). At the flagellar base, the basal body has nine outer microtubule triplets linked by the quintessential SAS6 nonamer, observed as a ‘cartwheel’ in EM cross‐sections (reviewed (Vaughan & Gull, 2015)). Transitional fibres connect the basal body microtubules to the plasma membrane and act as docking sites for IFT particles before their injection into the flagellum (Buisson et al., 2013; Garcia‐Gonzalo & Reiter, 2017). EM cross‐sections through the basal body reveal several structures that, while not yet observed in metazoa, are conserved in other protists, such as a set of fibres linking the triplet microtubules (termed the 'acorn' [Cavalier‐Smith, 2021; Geimer & Melkonian, 2004; Vaughan & Gull, 2015] that may form the 'terminal' plate (termed the acorn‐V system by [Cavalier‐Smith, 2021]) and may play a role in IFT passage (Dute & Kung, 1978; Ounjai et al., 2013; Vaughan & Gull, 2015).

**Figure 3:**
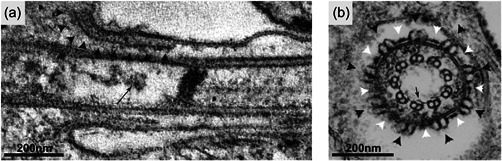
Structures of the transition zone. (a) A longitudinal section through the TZ reveals radial fibres that enter the TZ underlying the TZ membrane (arrowheads) and a filamentous structure in the TZ lumen (arrow). (b) A transverse TEM section reveals the external collarette made up of doublet‐tubules (black arrowheads) connected by fibres (white arrowheads). Fibrous ‘hooks’ penetrate into the lumen from the TZ doublets (arrow). Figure adapted from (Lacomble et al., 2009). TEM, transmission electron microscopy.

The transition zone (TZ) extends for 350 nm adjacently distal to the basal body and is comprised of both conserved and apparently parasite‐specific architecture (see [Cavalier‐Smith, 2021] for in‐depth overview of TZ structural conservation). As with TZs of most ciliated species (Barker et al., 2014), the TZ is a linked to the overlying membrane by Y linkers thought to act as a membrane diffusion barrier (Vaughan & Gull, 2015) and flagellar plaques observed in freeze‐fracture EM (Gadelha et al., 2009; Lacomble et al., 2009). However, several other trypanosome TZ structures have not been observed outside kinetoplastids, such as the collarette (observed as nine external double tubular structures connected by a fibrous sheath), a set of nine radial fibres underlying most of the TZ membrane, an electron dense filamentous structure in the TZ lumen, and short fibrous ‘hooks’ that extend from the A tubule towards the TZ lumen (Lacomble et al., 2009; Trépout et al., 2018) (Figure 3). The protein composition of the trypanosome TZ include orthologues of known ciliopathy complexes, such as Meckel‐Gruber syndrome and some components of nephronophthisis  (Barker et al., 2014), and most have been shown to have long term (days), stable association with the TZ (Dean et al., 2016). However, the majority of the 74 proteins that localise to the trypanosome TZ have no orthologues outside kinetoplastids and their function is not known (Dean et al., 2016).

The axoneme is formed by the extension of the TZ outer doublets and contains the archetypal structures associated with motility. Dynein motors and associated proteins are arranged in repeating units of 96 nm periodicity along the A tubule and are crucial for providing the mechanochemical energy for driving flagellar beating. Canonical features of these units include the outer and inner dynein arms, nexin dynein regulatory complex (NDRC) inter‐doublet linkages, and radial spokes that extend into the axoneme lumen. Recent elegant cryogenic electron tomography (cryoET) also revealed sets of apparently trypanosome‐lineage‐ specific microtubule inner proteins (MIPs) inside the A and B tubules that may stabilise them against forces generated as the trypanosome swims, or possibly provide a mechanism for fine‐tuning the beating of the axoneme (Imhof et al., 2019). Novel connections between the doublet microtubules were also observed that might facilitate doublet–doublet communication. Dynein arms are coordinated by the radial spokes and a central pair of microtubules that runs parallel to the outer doublets inside the axonemal lumen (Dawe et al., 2007; Hutchings et al., 2002; Ralston et al., 2006; Vaughan et al., 2006). The trypanosome central pair contains orthologues of known ciliopathy genes, such as Hydin and PF16, and mutants have the expected motility defects (Branche et al., 2006; Dawe et al., 2007; Ralston et al., 2006), suggesting similar mechanisms of flagellar beating. In contrast to *Chlamydomonas* and *Paramecium*, but comparable with some metazoa, the central pair has fixed orientation within the axoneme during flagellar beating and does not rotate (Branche et al., 2006; Gadelha et al., 2009; Mitchell, 2003; Omoto & Kung, 1979; Ralston et al., 2006; Sale, 1986; Tamm & Tamm, 1981). As in human tracheal cells (Saito et al., 2021), the trypanosome central pair is nucleated by the basal plate at the TZ‐axonemal boundary (Dean et al., 2019), in which it remains anchored (Höög et al., 2014). Only two components of the trypanosome basal plate are known, basalin and TZP103.8, and both are essential for central pair nucleation (Dean et al., 2019). Interestingly, basalin orthologues have extraordinarily high sequence divergence even between relatively closely related species, raising the prospect that it is broadly conserved in evolution; dissecting their mechanism of action will be key for understanding eukaryotic central pair assembly and motility.

### 3.1 The paraflagellar rod (PFR)—A sensory platform and/or a biomechanical spring?

The PFR is a Euglenozoa‐specific paracrystalline structure ~150 nm in diameter (Höög et al., 2012) that runs parallel to the axoneme (Bastin & Gull, 1999). It is present along the flagellum except at the flagellar pocket and ends shortly before the end of the flagellum and helps to coordinate the flagellar beat.

**Figure 4:**
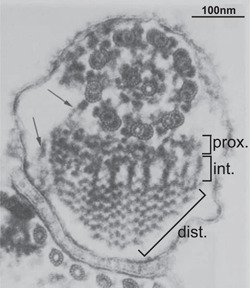
The PFR is an extra‐axonemal, structure required for flagellar beat regulation. A cross‐section through the flagellum reveals that the PFR is a paracrystalline structure with a proximal, intermediate, and distal domain, with respect to the cell body. The arrows indicate connections to an axonemal doublet and the flagellar membrane. Figure adapted from Sherwin & Gull (1989). PFR, paraflagellar rod.

The PFR is composed of three distinct domains which have been depicted by electron microscopy and defined by their position with respect to the axoneme: proximal, intermediate, and distal (Figure 4). Recent detailed cryogenic electron tomography (cryoET) (Zhang et al., 2021) revealed that the PFR is arranged in 54 nm repeating units along the longitudinal axis and is of triclinic paracrystalline form. Structures shaped like 'stacked scissors' (evident as 'comb teeth' in longitudinal sections) in the distal zone connect to form a stacked scissor density planar network (SSN), each comprised of five scissor stacks. SSNs are orientated at 45° to the axoneme axis and are connected by 'wires'. Density threshold analysis of SSNs suggests that ~90% of the PFR is 'empty space' that might be compressed during flagellum beating, and the PFR therefore utilise the principle of tensegrity, with elastic recoil potentially supported by the connections between the SSNs (Zhang et al., 2021). Therefore, the PFR may act as a biomechanical spring that disperses and transmits energy derived from axonemal beating (Bastin et al., 1998; L. C. Hughes et al., 2012; Koyfman et al., 2011).

The proximal domain attaches to the axonemal outer doublets 4–7 with direct connections to the outer arm dyneins on doublet 5 via PFR‐axoneme‐connectors (Zhang et al., 2021), while the distal domain is linked to the flagellum attachment zone (FAZ) (Höög et al., 2012; Vickerman, 1969), and contiguous structural connections between the distal, intermediate, and proximal zones that may allow signals to be propagated through the PFR (Zhang et al., 2021). Each domain is built of filaments of varying thicknesses and arrangements and with repeating units along the longitudinal axis (Höög et al., 2012; Koyfman et al., 2011; Portman & Gull, 2010; Vickerman, 1969).

Over 150 PFR proteins have been identified (Dean et al., 2017; Portman & Gull, 2010; Portman et al., 2009), of which PFR1 and PFR2 are the major structural components (Deflorin et al., 1994; Schlaeppi et al., 1989). RNAi mutant parasites of PFR2, one of the major PFR components, display motility defects that cause paralysis and sedimentation (Bastin et al., 1998), indicating that PFR has an essential role in motility, possibly through its connections with the outer dynein arms (L. C. Hughes et al., 2012; Zhang et al., 2021). The PFR has also been shown to play a role in motility by facilitating wave propagation and controlling cell shape (Branche et al., 2006). Calcium‐binding proteins like calmodulin have been found in the PFR and shown to associate with PFR1 and 2 proteins in a calcium‐dependent manner (Ridgley et al., 2000) surmising a possible role in beating cycle regulation. Other metabolic and signalling proteins, such as calcium‐binding proteins and cAMP‐dependant phosphodiesterases have been found in the PFR, inferring a possible role in intracellular signalling, and supporting motility through flagellar beat propagation (Oberholzer et al., 2006; Pullen et al., 2004; Ridgley et al., 2000). Mechanistic understanding of PFR function will be greatly advanced by ablating the plethora of PFR components and correlating protein identities with a detailed analysis of the resulting motility phenotypes and the loss of specific structures observed by cryoET.

### 3.2 The FAZ is a cellular ruler that defines cell morphology and cytokinesis

The flagellum is linked laterally to the cell membrane by the FAZ (Vickerman, 1969). The FAZ is a complex cytoskeletal structure comprised of large, interconnected fibres, filaments, and junctional complexes spread over up to 8 overlapping domains made up of 8 distinct structural zones and more than 20 different proteins (Lacomble et al., 2009; LaCount et al., 2002; Sunter, Benz, et al., 2015; Vaughan et al., 2008) (reviewed [Sunter & Gull, 2016]).

Of particular interest, the flagellar domain links the PFR and axonemal Doublet 7 to the flagellar membrane using fibres and junctional complexes (Höög et al., 2012; Vickerman, 1969). Several components are known and RNAi ablation of one member, ClpGM6, caused the entire FAZ to shorten and the flagellum basal body to move towards the cell's anterior pole, producing a morphological change reminiscent of lifecycle stages found in insect salivary glands. This implicates the FAZ as the master‐regulator of stage‐regulated shape and environmental habituation and it has therefore been termed the 'cellular ruler' (Hayes et al., 2014; Sunter, Benz, et al., 2015).

**Figure 5:**
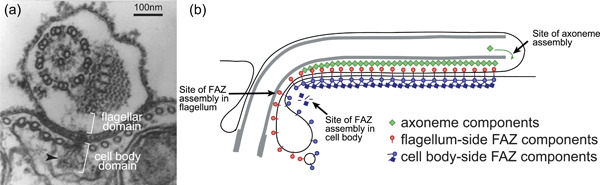
The FAZ has a complex cytoskeletal architecture. (a) A cross‐section through the trypanosome cell shows the FAZ domains, including the cell body and flagellar domains. The FAZ filament (arrowhead) links to junctional complexes on the plasma membrane. (b) A cartoon indicating the contrasting assembly sites of the FAZ (proximal) and axoneme (distal) necessitating that the different structures must move antagonistically during flagellum growth. Figure adapted from Sunter & Gull (2016). FAZ, flagellum attachment zone.

The cell body domain has a FAZ 'filament' that runs along the longitudinal axis of the FAZ and is visible as a series of fibres that radiate out from junctional complexes on the plasma membrane (Vaughan et al., 2008; Vickerman, 1969) (Figure 5). Ablation of FAZ filament proteins cause mispositioning of the basal body and misplacement of the cleavage furrow during cytokinesis, resulting in premature or asymmetrical cell division (Moreira et al., 2017). Other cell‐body FAZ RNAi phenotypes include either partial or complete flagellar detachment from the cell body, consistent with the FAZ's role in maintaining the flagellum‐cell body junction (LaCount et al., 2002; Nozaki et al., 1996; Vaughan et al., 2008).

In contrast to the incorporation of most flagellar components, which are conveyed to the distal tip by IFT trains, FAZ components are incorporated into the growing FAZ at the proximal end (Sunter, Varga, et al., 2015), meaning there must be complex remodelling of the FAZ at the interface with the axoneme and PFR during flagellum growth (Figure 5). Whether the new FAZ is ‘pushed’ along the oppositely growing flagellum when components are installed into the FAZ at the distal end, or they are pulled along the flagellum by an unknown component, is currently unknown.

### 3.3 The flagella connector (FC)—A motility in the eukaryotic flagellum unrelated to flagellar beating (or IFT)

**Figure 6:**
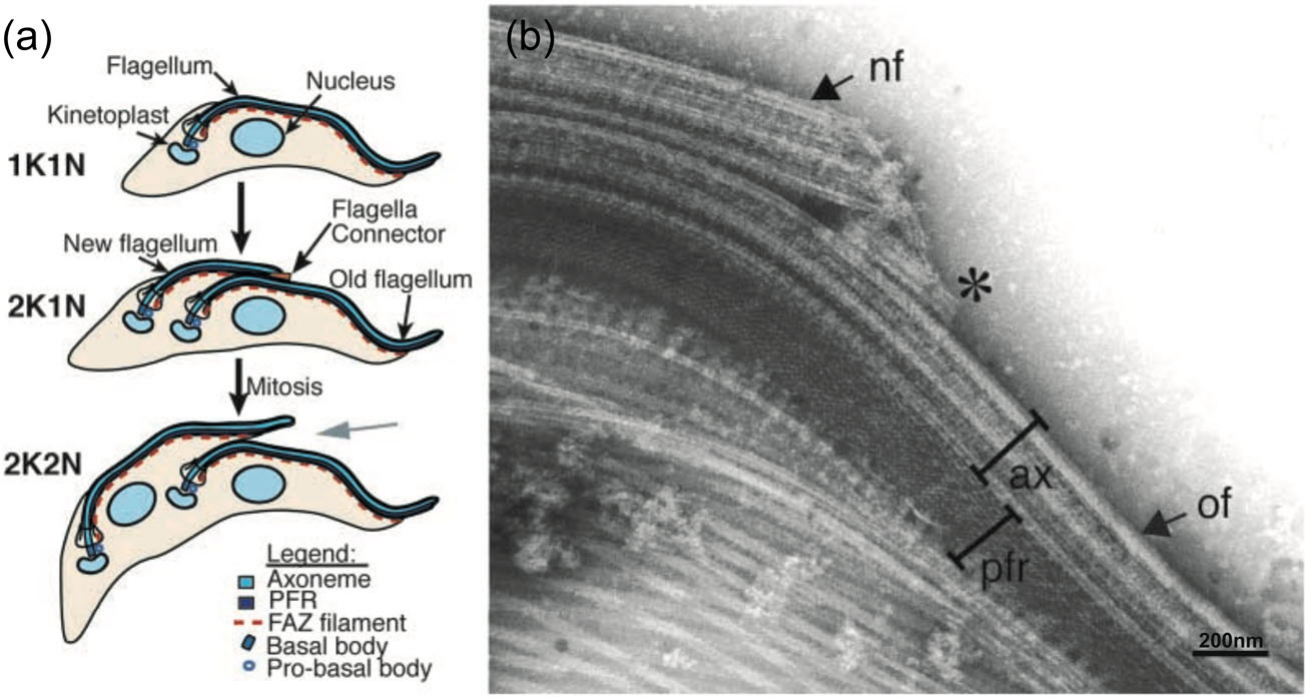
The flagella connector allows the old flagellum to template the path of the new flagellum. (a) The cell cycle of insect‐stage trypanosomes. At G1, trypanosomes possess a single kinetoplast and a single nucleus (1K1N) with a single attached flagellum. During the cell cycle, new flagellum elongation coincides with kinetoplast duplication and segregation (2K1N). The new flagellum is physically attached to the old flagellum via the flagella connector. Following mitosis (2K2N) and the initiation of cytokinesis, this connection is released. (b) Detergent treated cells ('cytoskeletons') were negatively stained and mounted whole onto grids for TEM. The flagella connector (asterisk) is positioned at the tip of the new flagellum (nf) and along the side of the axoneme (ax) of the old flagellum (of). Figure adapted from Briggs et al. (2004).

The FC is a motile cellular junction that bridges two flagellar membranes to link the microtubules of the tip of the new flagellum to the lateral microtubules in the old flagella axoneme (Briggs et al., 2004) (Figure 6). The FC is essential for cytokinesis to occur correctly and it defines the elongation path for the new flagellum using the physical connection to the old flagellum during the duplication cell cycle (Moreira‐Leite et al., 2001). The FC remains motile even when key IFT components are depleted (Kohl, 2003) causing extension of the new axoneme to cease; instead motility of the FC along the old axoneme is driven by FCP2/TbKinX1, a kinetoplastid‐specific kinesin that attaches the junction to the old flagellum axoneme (Varga et al., 2017).

The FC is formed quickly after the assembly of the TZ (Höög et al., 2016). FC movement along the old flagellum halts at a stop point ~0.6x the length of the old flagellum, which corresponds with the start of basal body migration and kinetoplast segregation (Davidge et al., 2006). Consequently, it has been hypothesised that the FC could play a role in basal body segregation by anchoring the tip of the new flagellum to the old flagellum during early flagellum elongation inside the flagellar pocket (Lemos et al., 2020). To date, the flagellar connector has only been observed in procyclic form of *T. brucei* during division in the assembly of a second flagellum (Höög et al., 2016).

The FC can be divided into five biochemically and structurally distinct zones (Briggs et al., 2004; Varga et al., 2017). Zone 1 links the new flagellum microtubule tips to Zone 2, which underlies the new flagellar membrane at the membrane junction. Zone 3 contains the membranes of both new and old flagellum at the junction and the interstitial layer and Zone 4 is the layer under the old flagellum membrane. Zone 5 is a filamentous network that links Zone 4 to microtubules of the old axoneme and contains FCP2/TbKinX1.

The FC is not present in the bloodstream form parasite. In this stage, the distal tip is contained in an invagination of the cell body plasma membrane termed the 'groove' associated with the FAZ, suggesting that it performs a similar role (L. Hughes et al., 2013). The relationship between the FC and the groove is not yet clear, however, the groove is proposed to have evolved to 'hide' the exposed proteins that link the old and new flagella in dividing bloodstream form cells from the host immune response (L. Hughes et al., 2013).

## 4 FLAGELLUM FUNCTION

### 4.1 Swimming between niches

**Figure 7:**
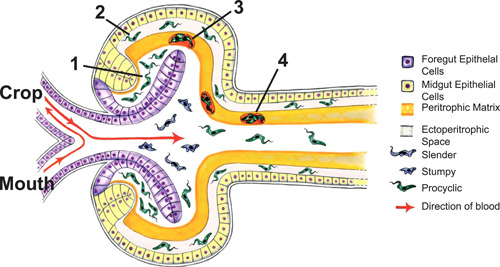
Trypanosomes must traverse the tsetse anatomy to infect new hosts. Upon inoculation, bloodstream form trypanosomes (blue cells) must first colonise the midgut and differentiate to insect procyclic forms (green cells) at the tsetse proventricular lumen (1). They then swim through the peritrophic matrix at the proventriculus to the ectoperitrophic space (2). Some cells become trapped in peritrophic matrix 'cysts' (3) which flow through the tsetse digestive tract (4) and are defecated. After colonising the proventriculus, the parasites swim to the salivary glands where they attach and then reinoculate new hosts. Illustration originally designed by Laura Jeacock and adapted from Rose et al. (2020).

Trypanosomes are digenetic parasites, requiring a tsetse fly vector and a mammalian host. There are seven different developmental forms that have been defined to date, each of which have specific adaptations for different niches in the host and the vector. There are two trypanosome morphological types, dependant upon where the flagellum is positioned in relation to the nucleus: ‘trypomastigote’, where the flagellum emerges from the cell posterior to the nucleus, and ‘epimastigote’, where the flagellum is positioned anterior to the nucleus (Vickerman et al., 1988). During the different lifecycle stages, the dimensions of the cell body, flagellum attachment to the cell body, and5 the free flagellum change. The insect stages are longer and thinner possibly as an adaptation for swimming between environments and swim with greater velocity to travel from the tsetse midgut to the salivary glands via the ectoperitrophic space and proventriculus (Figure 7). This contrasts with the bloodstream form of the parasite, which is adapted to overcoming blood components in the mammalian host (Bargul et al., 2016; Schuster et al., 2017; Wheeler, 2017). *Trypanosoma carassii* have shown different swimming techniques depending on the environment they are found in: in larger vessels they appear to actively swim against blood flow whereas in smaller capillaries they are dragged passively (Dóró et al., 2019). Flagellum function in trypanosomes is also essential for mammalian host infection, as parasites with dysfunctional LC1, a dynein subunit required for flagellar motility, cannot infect hosts (Shimogawa et al., 2018). Inducing RNA interference (RNAi) knockdown of flagellar proteins is lethal in the bloodstream form of the parasite (Broadhead et al., 2006; Kohl, 2003; Ralston & Hill, 2006).

## 5 IMMUNE EVASION

**Figure 8:**
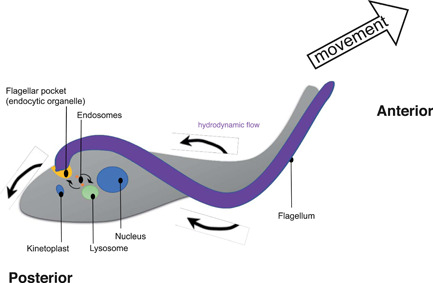
Hydrodynamic flow facilitates surface‐bound antibody clearance. As trypanosomes swim, hydrodynamic forces sweep surface‐bound antibodies to the cell's posterior. Antibody‐VSG complexes are internalised by endocytosis and the VSG is recycled to the cell surface after antibody removal in the endosomal system. Figure redrawn from Dean & Matthews (2007).

Trypanosome live extracellularly in host tissues and are exposed to the immune system. In order for them to evade host immune attack, the parasites have a variant surface glycoprotein (VSG) coat on the surface of their plasma membrane (Vickerman, 1969) that provides a barrier between the extracellular milieu and the plasma membrane that protects the cell from the innate and adaptive immunity (Vickerman, 1978). Immunoglobulin‐VSG complexes are endocytosed at a specialised invagination of the cell surface called the 'flagellar pocket' at the posterior end of the cell and the VSG is recycled to the cell surface after antibody removal in the endocytic system (Engstler et al., 2004; Grünfelder et al., 2003). Parasite motility through blood has been proposed to give rise to hydrodynamic flow that ‘sweeps’ surface‐bound antibodies to the flagellar pocket, facilitating their removal from the cell surface (Figure 8), especially in the key early infection when the antibody titre is relatively low (Dean & Matthews, 2007; Engstler et al., 2007). According to this model, if surface‐bound antibodies are not removed, macrophages recognise and phagocytose the parasite and clear the infection (Cheung et al., 2016).

## 6 CELLULAR FUSION AND ADHESION TO SURFACES

The trypanosome life cycle involves several key changes including meiosis and genetic exchange and can take up to 3 weeks. For trypanosome reproduction to occur, the parasite's flagellar membrane attaches to the brush border of the salivary gland epithelium (Gibson et al., 2008) of the tsetse fly. This attachment is mediated by intricate outgrowths and hemidesmosome‐like attachment plaques (Tetley & Vickerman, 1985; Tetley et al., 1987; Vickerman et al., 1988). The trypanosome flagellum has also been suggested to have a role bringing gametes into close proximity (Peacock et al., 2014) and has been shown to mediate cell–cell fusion in other lifecycle stages, although the purpose of this mechanism is still unknown (Imhof et al., 2016).

### 6.1 The trypanosome flagellum is a sensory organelle

Flagella have well‐established roles in sensing and chemotaxis in higher eukaryotes (Bloodgood, 2010). Evidence suggests that this is also the case in African trypanosomes and a range of sensory molecules appear to concentrate in the flagellum.

Adenylate cyclases (ACs) are important signalling proteins that produce the secondary messenger cyclic adenosine monophosphate (cAMP), often in response to external stimuli. Trypanosomes have over 80 ACs (Salmon et al., 2012), several of which are found in the flagellum and the flagellar membrane and appear to aid in host immune response evasion and mediate insect tissue distribution and coordinated motility (Durante et al., 2020; Lopez et al., 2015; Salmon, 2018; Seebeck et al., 2004). Some flagellar ACs are upregulated in the procyclic form of the parasite, suggesting a stage‐specific role, and are characterised by glycosylation, catalytic activity and being dimerising surface‐exposed proteins (Saada et al., 2014). Expression site associated gene 4 (ESAG4) encodes one of several transmembrane ACs located in the trypanosome flagellar membrane and ESAG4 mutagenesis causes a reduction of the parasite control of early host immune response through tumour necrosis factor‐α (TNF‐α) synthesis inhibition in liver myeloid cells (Paindavoine et al., 1992; Salmon et al., 2012).

Unicellular organisms exchange chemical cues similar to a multicellular entity, such as biofilm formation, social motility, fruiting body development and quorum sensing. When insect stage trypanosomes are cultured on agarose plates at high densities, they engage in a coordinated movement termed social motility, or SoMo, which has become a proxy for cell–cell communication (Imhof et al., 2014; Oberholzer et al., 2010). Flagellar cAMP signalling appears to play a key role in trypanosome SoMo. Inhibiting the flagellar‐bound cAMP‐specific phosphodiesterases B1 (PDEB1) in trypanosomes, which hydrolyses cAMP, causes increased intracellular cAMP levels, abolishes pH‐ taxis (Shaw et al., 2021) and blocks SoMo, without impacting the viability or motility of individual cells (Oberholzer et al., 2015). Importantly, PDEB1 null mutants are trapped in the lumen of the tsetse midgut, demonstrating that flagellar cAMP signalling is essential for crossing the peritrophic matrix and completing the lifecycle in the insect (Shaw et al., 2019). Two other flagellar‐bound sensory proteins, adenylate cyclase 5 (ACP5), and cAMP response protein 3 (CARP3) also appear to be involved in the response to acid and RNAi mutants show a major SoMo defect similar to PDEB1 knockout phenotype (Shaw et al., 2021), whereas ablation and dominant‐negative mutants of AC6, and dual ablation of AC1 and AC2, cause a hyper‐social phenotype (Lopez et al., 2015). Cyclic nucleotides have already been proposed to regulate microbial social behaviour and this evidence suggests that this is also true in African trypanosomes.

FLAgellar Member 8 (FLAM8), a flagellar membrane‐bound protein, is required for extravascular *Trypanosoma brucei* parasite dissemination in mammalian host tissues, especially in the skin (Alvarez et al. 2020, 2021). Although its biochemistry and mechanism of action is not yet clear, to date this is the only example of a protein that influences parasite distribution in host tissues and may indicate a role for the flagellum in host tissue tropism.

## 7 IS THE TRYPANOSOME FLAGELLUM DRUGGABLE?

Sustained research over many years has resulted in powerful tools to aid the development of drugs against human and animal trypanosomiases. One of the most important is the development of genome‐wide RNAi libraries (Alsford et al. 2011, 2012; Baker et al., 2011; Mackey et al., 2011; Schumann Burkard et al., 2011; Stortz et al., 2017) that has identified the genes that are essential in the pathogenic lifecycle stage, allowing genome‐scale discovery of potential drug targets.

Similarly, whole‐genome gain‐of‐function libraries (Begolo et al., 2014; Carter et al., 2020) have been deployed to identify drug resistance mechanisms that might occur in the field. Combined with powerful genetic tools for functional analysis, such as inducible RNAi, CRISPR knockouts, and inducible ectopic‐expression, gene‐function can be rapidly addressed, allowing the design of combination therapies to target different pathways, reducing the risk of resistant strains being selected.

The trypanosome flagellum is ripe for development as a drug target. Multiple flagellum proteomics studies on different lifecycle stages and fractions (Broadhead et al., 2006; Oberholzer et al., 2011) combined with genome‐scale tagging (Dean et al., 2017; Dyer et al., 2016) have revealed that it is complex and contains many classes of protein that are ‘druggable’, such as adenylate cyclases, phosphodiesterases and kinases (discussed in more depth below), meaning that existing drug development pipelines and libraries can be more easily leveraged. Further, its ensemble of extra‐axonemal structures, such as the PFR and FC, suggest that there are parasite‐specific processes that can be specifically targeted, reducing the risk that anti‐flagellum chemotherapies may have off‐target effects. Indeed, the lineage‐specific elaborations of dyneins in trypanosomes reported by Imhof et al. (2019) may present additional druggable targets because dynein inhibitors with isoform specificity have now been developed (Firestone et al., 2012; Reddy et al., 2017; See et al., 2016). A number of studies have shown that the flagellum is essential for parasite viability. In the host, flagellum function is essential for virulence and functional impairment allows clearance of the infection by the host immune system (Shimogawa et al., 2018). However, although RNAi studies demonstrate that the flagellum is essential even in axenic culture (Alsford et al., 2011; Branche et al., 2006; Broadhead et al., 2006; Ralston & Hill, 2006; Ralston et al., 2011), milder motility defects that retain flagellar structures appear to be somewhat tolerated (Kisalu et al., 2014; Ralston et al., 2011). This highlights a difference between RNAi mutants, which may involve the loss of multiple proteins and structures from the cytoskeleton, and the expression of mutant proteins, which may more closely resemble the effect of an inhibitor.

The trypanosome flagellum is an important site of cAMP signalling and harbours adenylate cyclases (cAMP catabolism) and phosphodiesterases (cAMP hydrolysis) and cAMP response proteins (CARPs) (Alexandre et al., 1990; Paindavoine et al., 1992). Simultaneous inhibition of flagellar phosphodiesterases PDEB1 and PDEB2 increases cellular cAMP, causing cell death and preventing mouse infection, suggesting this may be a viable strategy for chemotherapy. Expression of a dominant‐negative flagellar AC reduced surface AC activity by ~50%, stopping trypanosome inhibition of the innate immune response (Salmon et al., 2012), suggesting that targeting surface adenylate cyclases could inhibit trypanosome immune evasion.

Kinases are highly druggable enzymes and more than 100 kinase inhibitors are currently in clinical trial, several of which have been shown to have potent ant‐trypanosome properties (Brimacombe et al., 2014; Diaz et al., 2014; Urbaniak et al., 2012). Approximately, 2% of the trypanosome genome encodes for kinases, including 1 class of atypical kinases, suggesting that kinases may be a good drug target in trypanosomes (Merritt et al., 2014). Trypanosome kinases localise to different cellular compartments, including the different flagellar structures, and have diverse roles in cell division and cellular signalling (Oberholzer et al., 2011). Several studies have shown that flagellar kinases, including the basal body, PFR and axoneme, are essential for cell viability in vitro and in vivo, suggesting they may be a good drug target that can be exploited for infection control (Dean et al., 2017; Fernandez‐Cortes et al., 2017; Jones et al., 2014; Mackey et al., 2011).

Although phosphatase inhibitor drugs have traditionally been difficult to develop due to their charged active site biochemistry, several are now in clinical use (Mullard, 2018). The trypanosome genome encodes at least 78 phosphatases with almost 500 phosphoproteins (Brenchley et al., 2007; Szöör, 2010). Although the function of most are not well understood, a genome‐wide screen determined that serine threonine‐protein phosphatase PP1‐6, that localises to the basal body and axoneme (Dean et al., 2017), may be involved in trypanosome lifecycle differentiation (Mony et al., 2014) and KKP1 was recently shown to be essential for flagellum duplication and cell viability (An et al., 2021).

Notwithstanding the considerable practical hurdles, at least in principle a drug that targets trypanosome transmission via tsetse would be a powerful tool in the fight to eliminate African trypanosomiasis and reduce the burden of disease in cattle. The flagellum's role as a sensory organelle is key to its traversal through the tsetse, and genetic inhibition of PDEB1 causes loss of SoMo and progression through the tsetse (Oberholzer et al., 2015; Shaw et al., 2019). Flagellar motility and forward directionality is required for the parasite to reach the foregut and complete their lifecycle and several flagellar motility mutants have been shown to be ‘stranded’ in the tsetse midgut and unable to traverse to the salivary glands, possibly due to their inability to cross the peritrophic matrix (Rotureau et al., 2014), meaning a drug that targets flagellar mechanochemistry would stop transmission. Similarly, although its precise role is not clear, the flagellar localised arginine kinase is important for tsetse colonisation (Ooi et al., 2015). More recent reports raise the possibility that the flagellum may play roles in host tissue tropism, and targeting this function could impact their transmission efficiency (Alvarez et al., 2021).

## 8 CONCLUSION

Trypanosomes are an extraordinary powerful model system for understanding eukaryotic biology. As an early diverging (but highly sophisticated) protist, their biology reflect traits found in the last eukaryotic ancestor or constraints upon eukaryotic cell biology and therefore provides insights into the 'rules' of eukaryotic biology. Their sometimes 'extreme' biology combined with their superb experimental tractability mean that they have contributed greatly to the early characterisation of conserved biology, such as glycosylphosphatidylinositol  anchors, RNAi and RNA editing. Moreover, its infectious disease relevance and its potential for providing greater insights into human genetic diseases of the cilia (ciliopathies) mean that the trypanosome is an exceptional system for investigating the eukaryotic flagellum.

## CONFLICT OF INTEREST

The authors declare no conflict of interest.
